# Insulin Resistance and Oxidative Stress in the Brain: What’s New?

**DOI:** 10.3390/ijms20040874

**Published:** 2019-02-18

**Authors:** Mateusz Maciejczyk, Ewa Żebrowska, Adrian Chabowski

**Affiliations:** Department of Physiology, Medical University of Bialystok, Mickiewicza 2c Str., 15-222 Bialystok, Poland; ewa.zebrowska13@gmail.com (E.Ż.); adrian@umb.edu.pl (A.C.)

**Keywords:** brain, insulin resistance, oxidative stress, mitochondrial dysfunction

## Abstract

The latest studies have indicated a strong relationship between systemic insulin resistance (IR) and higher incidence of neurodegeneration, dementia, and mild cognitive impairment. Although some of these abnormalities could be explained by chronic hyperglycaemia, hyperinsulinemia, dyslipidaemia, and/or prolonged whole-body inflammation, the key role is attributed to the neuronal redox imbalance and oxidative damage. In this mini review, we provide a schematic overview of intracellular oxidative stress and mitochondrial abnormalities in the IR brain. We highlight important correlations found so far between brain oxidative stress, ceramide generation, β-amyloid accumulation, as well as neuronal apoptosis in the IR conditions.

## 1. Introduction

Nowadays, one of the most significant health problems is an increased incidence of obesity and type 2 diabetes (T2DM) [1,2,3]. A number of epidemiological and clinical research studies have indicated that overnutrition, low physical activity, as well as many genetic and environmental factors (e.g., chronic stress, xenobiotics, and alcohol abuse) are the main causes of metabolic disorders referred to as “the epidemics of the 21st century” [1,2,3]. T2DM, the most common type of diabetes mellitus, is generally characterized by chronic hyperglycaemia, hyperinsulinemia, dyslipidaemia, as well as lipotoxicity, which result in progressive deterioration of insulin secretion and insulin action [3,4,5,6]. It is widely recognized that insulin resistance (IR) plays a key role in the development of T2DM and its organ-related complications [5,6]. Thus, an explanation of IR pathogenesis is of great clinical significance.

Metabolic disturbances accompanying IR disrupt not only the functioning of liver, muscles and adipose tissue, but also the brain [7,8,9,10,11]. Although for many years the brain has been considered an insulin-insensitive organ, it is now known that insulin acts a critical role in the central nervous system (CNS) participating in neuronal survival, neuroplasticity, memory, and cognitive functions [10,12,13,14]. Additionally, recent studies have demonstrated that peripheral IR results in loss of the brain function, which indicates strong relationship between metabolic disturbances and cerebral degeneration, cognitive impairment, depression, as well as Alzheimer’s disease (AD) [7,9,15,16,17,18]. It is suggested that one possible common denominator of all these conditions could be chronic oxidative stress [7,8,9,15,16,17,18]. Indeed, in various systemic and genetic diseases, alterations in enzymatic and non-enzymatic antioxidant systems as well as increased oxidation of cellular components are the main factors damaging the brain tissue and promoting cerebral degeneration [19,20,21,22,23]. Nevertheless, the exact role of brain oxidative damage and free radical sources in the brain IR are still unknown. Hence, in this mini review, we provide an overview of the neuronal redox imbalance, oxidative damage, as well as mitochondrial dysfunction in IR conditions. Mechanistic insights between the brain IR, ceramide generation, β-amyloid accumulation, as well as neuronal apoptosis are also presented.

## 2. Insulin Role in the Brain

Insulin, an anabolic peptide hormone, plays a crucial role in the regulation of whole-body glucose homeostasis. Most insulin receptors are located in the target tissues (e.g., liver, skeletal muscles, and adipose tissue), however, their high cellular density has also been reported in the brain, mainly in the areas responsible for regulation of cognitive function, appetite, as well as autonomic activity [10,12,14,24]. Interestingly, insulin in the brain may origin not only from pancreatic β-cells, but also can be synthesized *de novo* in the neurons and glial cells [12,14,25]. Nevertheless, regardless of insulin action/production site, this hormone exerts a similar molecular mechanism both in the brain and in the periphery [7,26,27]. As demonstrated in numerous studies, insulin action on the brain includes food intake regulation, feeding behavior, body weight, as well as energy homeostasis [12,14,24,27]. These effects may be mediated by two major components of the brain insulin transduction systems: phosphatidylinositol-3-kinase (PI3K)/Akt pathway and mitogen-activated protein kinases/Ras pathway (MAPK/Ras) [12,28]. However, some of insulin actions are specific for the CNS. Indeed, insulin has several neuronal roles: provides neuronal survival, participates in synaptic plasticity, and regulates the brain functioning including memory, cognition, learning, as well as attention [14,26,29,30]. It was shown that insulin can modulate neuronal activity through various molecular mechanisms [12,14,31,32]. This hormone affects neurotransmitter receptor density, inhibits norepinephrine and stimulates serotonin reuptake in the CNS synapses [12,33]. It also modulates long-term potentiation (LTP) and long-term depression (LTD) by reducing the amount of AMPA (α-amino-3-hydroxy-5-methyl-4-isoxazolepropionic acid) receptors for glutamate as well as by stimulating the translocation of GABA (γ-aminobutyric acid) receptors in the post-synaptic membrane [12,31,32].

It is postulated that insulin may also participate in neuronal glucose metabolism. Although the insulin-regulated glucose transporter GLUT-4 has been identified in the brain [34], it remains an open question whether its translocation is dependent on the insulin action. In recent studies, NIRKO (neuronal insulin receptor knockout) mice showed alterations in glucose-sensing hypothalamic neurons in response to hypoglycemia [35]. Studies conducted on brain GLUT-4 knockout (BG4KO) mice also suggest an important role of GLUT-4 in the regulation of systemic glucose level [36]. Nevertheless, there is no convincing evidence that transport and utilization of neuronal glucose is regulated by the insulin-mediated pathways. However, it should be emphasized that, regardless of the plasma insulin level, cerebral insulin also determines many metabolic effects via modulation of the vagal and sympathetic efferent fibres [37]. These actions include suppression of hepatic glucose production, hepatic triglyceride secretion, as well as lipolysis in the adipose tissue [26,30,37].

## 3. Brain Insulin Resistance

A crucial role in T2DM pathology is attributed to insulin resistance. IR is defined as the lack or decreased response of the target tissues to insulin [38,39]. At the molecular level, IR is caused by a loss/down regulation of the insulin receptors and insulin receptor substrates (IRS-1 and IRS-2), as well as by impairment of the insulin receptor’s binding activity [5,38]. Functionally, reduced brain sensitivity to insulin can manifest as alterations in neurite outgrowth, impaired neuroplasticity and disturbances in neurotransmitter’s release and uptake [10,16,40]. Bearing in mind that many factors contribute to insulin transport to the brain (e.g., lipotoxicity, glucotoxicity, inflammation, and oxidative stress), systemic IR may affect the cerebral insulin signalling as well as lead to impairment of the insulin-induced LTD [16,29,41,42]. Indeed, it has been demonstrated that peripheral IR (or high circulating insulin level) alters the function of the blood-brain barrier (BBB) by reducing the level of endothelial insulin receptors and decreasing the BBB permeability to insulin [43,44]. This results in the impairment of physiological insulin functions as well as increased BBB permeability to many substances [26,27].

Although many causes of cerebral IR have been proposed to this day, the only confirmed explanation is ceramide accumulation in the brain tissue [11,16,45,46]. Ceramide, a compound of amino alcohol sphingosine and a long saturated fatty acid (C:16-C:32), belongs to a large group of biologically active sphingolipids that build cell membranes of neurons and glial cells [47,48]. In addition to the structural properties, ceramide participates in the growth, differentiation, proliferation, and aging of these cells [47,49]. However, large amounts of this compound are also produced in the liver. It was shown that peripheral IR is associated with an elevated ceramide generation due to the increased supply of fatty acids (FAs) derived from a high fat diet (HFD) [50,51,52]. Interestingly, ceramide like other neurotoxic lipids passes through the BBB, contributing to the brain IR via liver-brain axis of neurodegeneration [16,45,46,53]. Indeed, several studies have indicated that increased ceramide synthesis in the liver associated with diet-induced IR may also mediate cerebral IR [8,16,41,45,54]. Interestingly, in an animal model of IR, reduced insulin sensitivity in the brain confirmed by decreased phosphorylation of insulin receptors/substrates as well as downstream insulin signalling (↓PI3K-Akt pathway) was reported after different periods of HFD administration (i.e., 5–12 weeks) [9,41,55,56]. These differences may result from various diet compositions as well as varied animal models used in the experiments [9]. Moreover, ceramide can also be produced in the brain (*via de novo* synthesis and/or sphingomyelin hydrolysis). Elevated synthesis of neuronal ceramide associated with impaired insulin signalling was observed not only in vitro and in animal models of IR, but also in the brain of IR patients [8,41,57,58].

In physiological conditions, insulin binding of the insulin receptor activates insulin receptor substrate 1 (IRS-1), which in turn promotes PI3K, then Akt, and finally the downstream targets of Akt such as mTOR (mammalian target of rapamycin kinase), GSK3β (glycogen synthase kinase 3 beta), as well as FOXO (forkhead box transcription factors of the class O) [6,12,59]. However, in IR patients, insulin transmission is diminished [46,54,60,61,62]. It was shown that induction of cerebral IR results from ceramide-mediated phosphorylation of IRS-1 and inhibition of PI3K-Akt signalling in the brain [46,54,60,61,62]. Briefly, ceramide activates several serine-threonine kinases (e.g., c-Jun N-terminal kinases (JNKs) and IκB kinase (IKK)) able to phosphorylate serine and thus inhibits insulin receptors substrates (mainly IRS-1) [46,60,61,62]. Consequently, ceramide downregulates Akt phosphorylation and kinase activity via induction of protein phosphatase 2A (PP2A) as well as activates interleukin-1β converting enzyme (ICE)-like proteases, resulting in the disruption of insulin signalling pathway and promotion of neuronal apoptosis [16,54,62]. Additionally, it has been demonstrated that ceramide impairs brain cell viability, energy metabolism and mitochondrial activity, and therefore, may mediate neuro-cognitive deficits in IR patients [41,46,54,60,61,62]. Although mainly long chain ceramide (C24, C36) is able to induce cerebral IR, recent studies indicated that also short-chain ceramide (C2) may alter insulin transmission in the brain [45,54]. Additionally, it was shown that ceramide supresses anti-apoptotic Bcl-2 and inhibits protein kinases B (PKB) and C (PKC-α) [63,64]. Ceramide-induced apoptosis, similarly as in the neurodegenerative diseases, may therefore cause cerebral complications of IR [65,66].

Despite the proven role of ceramide’s neurotoxic effects in the brain, the pathogenesis of cerebral IR is still not well understood and required further research.

## 4. Alzheimer’s Disease as a Type 3 Diabetes

AD, the most common type of dementia, is characterized by gradual decline of memory and cognitive functions associated with accumulation of neurotoxic amyloid-β (Aβ) in the forebrain [67,68]. The phenotypic hallmark of AD also includes intraneuronal deposition of hyperphosphorylated and polyubiquitinated proteins (mainly tau protein) resulting in accumulation of neurofibrillary tangles (NFTs), neuronal loss, gliosis, dystrophic neuritis, as well as neuropil threads [10,67,68]. Although the exact causes of the disease are still not well understood, many studies indicate disturbed insulin signalling in the AD brain [10,18,29,69,70]. Nevertheless, alterations in the cerebral glucose utilization as well as defects in insulin transmission have been mainly reported in the early stages of Alzheimer’s disease [61,71,72]. De la Monte et al. [58,73] observed decreased mRNA/protein expression of insulin receptors, insulin-like growth factors (IGF1 and IGF2), IRS1, PI3K/Akt as well as increased GSK-3β level in the post-mortem brains from AD patients. Interestingly, the observed changes were inversely correlated with neuropathological features of AD, which indicates the relationship between impaired insulin signalling and progression of the disease [70]. Deficits in insulin/IGF receptor binding were also noted in the cerebrospinal fluid (CSF) of AD patients [58,70,74].

It has been demonstrated that many abnormalities in AD pathology (e.g., increased tau phosphorylation, disturbances in energy metabolism, neuronal growth as well as synaptic plasticity) may result from impaired insulin signalling in the CNS. Indeed, in AD brain, cerebral insulin/IGF resistance leads to increased activation of GSK-3β [70,75,76]. It was shown that stimulation of GSK-3β promotes hyperphosphorylation of tau protein, and therefore induces tau misfolding and fibril aggregation in the brain [70,75]. However, tau hyperphosphorylation is also mediated by up regulation of cyclin-dependent kinase 5 (cdk-5) and inhibition of PP2A [73,77]. Additionally, also Aβ peptides disrupt insulin transmission in AD brain by competing with insulin and/or reducing the binding affinity of insulin to its receptor [73,78,79]. It was shown that Aβ peptides directly interferes with PI3 kinase activation of Akt, leading to GSK-3β induction and associated tau pathology [73,78,79]. Finally, in AD brain, decreased insulin signalling through IRS, PI3K and Akt may also be linked to a loss in neuronal/oligodendroglial survival, cell growth, as well as neuroplasticity [61,73]. Since abnormalities in AD brain are characterized by both insulin resistance and insulin deficiency, it is proposed that Alzheimer’s disease could be referred as “brain diabetes” or “type 3 diabetes” [58,67,80].

The crosstalk between impaired insulin transmission and cognitive impairment may also be evidenced by benefits from intranasal insulin administration in patients with AD. Indeed, several studies have demonstrated that intranasal insulin treatment improves cognitive performance, attenuates hyperphosphorylation of tau protein, as well as ameliorates microglial activation in individuals with an early stage of AD [81,82,83,84].

## 5. Oxidative Stress in the Brain Insulin Resistance

The latest clinical studies have shown a strong relationship between whole body IR and higher incidence of neurodegeneration, dementia, depression, and mild cognitive impairment [7,9,15,16,17,18,46]. Although some of these abnormalities could be explained by chronic hyperglycaemia, hyperinsulinemia, dyslipidaemia, and/or prolonged whole body inflammation, the key role is attributed to the mitochondrial dysfunction and brain oxidative stress [7,8,9,15,16,17,18,85,86,87]. Indeed, oxidative stress is the main pathological factor responsible for metabolic abnormalities (e.g., disturbances in glucose and lipid metabolism), as well as cerebral degeneration, aging, and CNS injury [6,7,23,88]. Generally, oxidative stress is defined as an imbalance between the production of reactive oxygen (ROS) and nitrogen (RNS) species as well as the efficiency of enzymatic (e.g., catalase, glutathione peroxidase, superoxide dismutase) and non-enzymatic (e.g., reduced glutathione (GSH), uric acid) antioxidative systems [89,90]. Redox abnormalities in favour of oxidative reactions result in oxidative damage, wherein both proteins, lipids and nucleic acids are oxidized. The effect of free radical interactions may be damage to cell membrane or mitochondria, accumulation of oxidatively modified proteins, as well as the formation of genetic mutations in DNA/RNA [91]. High reactivity of free radicals results from their very short half-life as well as the ability to give/attach valence electrons, which participate in biochemical reactions. The resulting by-products of oxygen metabolism (including newly generated free radicals) react with all the encountered biomolecules [90,91]. Thus, the consequences of the ROS/RNS activity can occur at considerable distances from the site of the primary oxidative damage [92]. It is well known that oxidative modification products may disrupt cell metabolism and signalling, in particular increasing the production of pro-inflammatory cytokines, modifying gene expression, and promoting cell death by apoptosis and necrosis [90,91].

**Figure 1:**
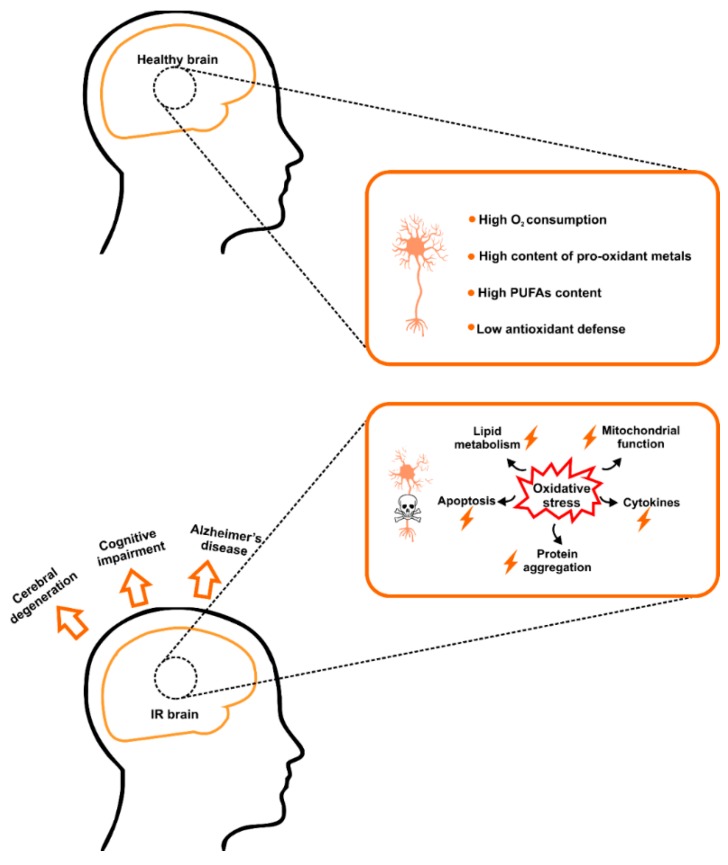
The brain as a specific target for insulin resistance and oxidative stress. The brain is particularly sensitive to the free radical attack due to its increased oxygen consumption, limited antioxidative mechanisms, as well as high levels of polyunsaturated fatty acids (PUFAs). In IR brain, overproduction of reactive oxygen species (ROS) leads to oxidative damage associated with ATP depletion, activation of pro-inflammatory cytokines, accumulation of protein aggregates, as well as neuronal apoptosis. Abbreviations: IR, insulin resistance; PUFAs, polyunsaturated fatty acids.

Under IR conditions, disturbances in enzymatic and non-enzymatic antioxidants as well as the increased content of oxidative modification products have been reported in serum/plasma [93,94,95,96,97] as well as liver, muscles, adipose tissue, and brain tissue [9,86,93,96,97,98,99]. However, of all body organs, the brain is particularly sensitive to the free radical attack [100,101]. Constituting less than 2% of the body, the brain uses over 20% of the oxygen delivered to the organism. Phospholipids of cerebral cell membranes are also enriched in polyunsaturated fatty acids (PUFAs) which, together with the low activity of brain antioxidant enzymes and the high content of pro-oxidant metal ions (e.g., Fe^2+^, Cu^2+^, Co^2+^, and Cr^2+^), make the brain very vulnerable to oxidative stress [93,100,101]. Therefore, this is not surprising that in the IR brain, overproduction of ROS leads to oxidative damage associated with higher cell membrane permeability, ATP depletion, and accumulation of protein aggregates. One of the deleterious consequences of lipid and protein oxidation may also be an induction of pro-inflammatory enzymes and thus stimulation of the brain inflammation [7,8,9,67,99,102,103]. The abovementioned, all together, may predispose neurodegeneration and/or neuronal apoptosis (Figure 1).

As demonstrated in recent studies, increased efficiency of enzymatic (e.g., ↑catalase, ↑superoxide dismutase, ↑glutathione peroxidase, ↑glutathione reductase) and non-enzymatic (↑uric acid) brain antioxidants observed in IR conditions should be considered as an adaptive reaction in response to enhanced free radical production [55,93,104,105]. Indeed, it is well-known that strengthening the antioxidant barrier is the basic protective mechanism against the ROS-mediated injury [106]. However, such changes have not been observed in the cerebral glutathione (GSH) level [55,107,108]. Interestingly, lowered GSH levels (with a simultaneous increase in GSSG (oxidized glutathione) levels) were reported not only in the IR brain, but also in patients with AD, Parkinson’s disease, multiple sclerosis, amyotrophic lateral sclerosis and Huntington’s disease [55,107,108,109,110,111,112]. It is postulated that disturbances in glutathione metabolism may result in the impairment of cerebral functioning in both IR and neurodegenerative diseases [110,111]. This fact is not surprising because GSH is the most important of the brain’s antioxidants [112,113]. This compound, in addition to the antioxidant properties, also participates in the regeneration of other free radical scavengers (e.g., vitamins C and E), regulates gene expression (including the insulin signalling proteins), maintains sulfhydryl groups (-SH) in the reduced state, as well as affects proliferation, differentiation and neuronal apoptosis [109,111,112,114]. However, also advanced glycation end products (AGE, products of glycation and oxidation by reducing sugars) are an important link between IR and cerebral degeneration [115,116,117]. It has been demonstrated that the accumulation of protein aggregates leads to morphological changes in the brain tissue and increases the production of pro-inflammatory cytokines and chemokines [115,118,119,120]. Moreover, it has been shown that AGE may increase production of free radicals by inducing the activity of NADPH oxidase (NOX), which is the main source of ROS in neurons and glial cells [116,121,122]. Indeed, the increased AGE content was demonstrated in patients with IR and neurodegenerative diseases, especially in the hyperglycemic conditions [93,119,120,123,124]. Nevertheless, different cerebral structures are characterized by varied sensitivity to the redox imbalance and oxidative stress [93,102]. As demonstrated in recent studies, the cerebral cortex of IR rats is more strongly affected by oxidative stress than the hypothalamus [93]. The dissimilar response of the brain structures may result from changes in mitochondrial bioenergetics as well as distinctive ability to accumulate pro-oxidant metal ions (mainly Fe^2+^ and Cu^2+^) [125,126].

## 6. Metabolic Disturbances as a Link between Brain Insulin Resistance, Oxidative Stress and Neurodegeneration

**Figure 2:**
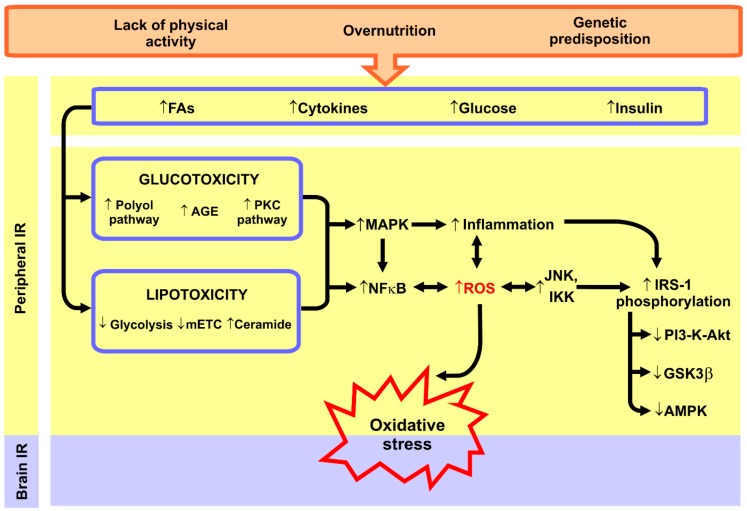
Metabolic disturbances in peripheral insulin resistance as a source of brain oxidative stress**.** Under insulin resistance (IR) conditions, enhanced plasma fatty acids (FAs), hyperglycaemia, and hyperinsulinemia promote glucotoxicity and lipotoxicity (especially ceramide accumulation) leading to the activation of nuclear factor κB (NFκB) and pro-inflammatory signalling (e.g., MAP-kinases). Not only does it intensify the inflammation, but it also increases the formation of reactive oxygen species (ROS). Abbreviations: AGE, advanced glycation end products; AMPK, AMP-activated protein kinase; FAs, fatty acids; GSK3β, glycogen synthase kinase 3 beta; IKK, IκB kinase; IRS-1, insulin receptor substrate 1; IR, insulin resistance; JNK, c-Jun terminal kinase; MAPK, mitogen-activated protein kinases; mETC, mitochondrial electron transport chain; NFκB, nuclear factor κB; PKC, protein kinase C; PI3-K-Akt, phosphatidylinositol 3-kinase/Akt; ROS, reactive oxygen species.

According to the current state of knowledge, metabolic disorders accompanying peripheral IR are thought to induce neuronal oxidative stress. It is well established that in insulin-sensitive peripheral tissues, excessive intracellular lipid accumulation (mainly diacylglycerol (DAG) and ceramide) inhibits insulin signalling pathways leading to decreased glucose utilisation [5,38,127,128]. In these tissues, the production of pro-inflammatory cytokines (e.g., IL-1β, IL-6, TNF-α) also increases, which activates several serine-threonine kinases (e.g., JNK and IKK) phosphorylating the serine residues of the insulin receptor substrate 1 (IRS-1), which in turn blocks the insulin signalling proteins (e.g., PI3-K-Akt, GSK3β, and AMPK) [6,129,130,131]. Additionally, it has been demonstrated that the toxic effects of hyperglycaemia are associated with the activation of the polyol/protein kinase C (PKC) pathway, autooxidation of glucose, as well as accumulation of AGE [5,128,132,133], whereas the excessive availability of FAs inhibits glycolysis and impairs the functioning of the mitochondrial respiratory chain (mETC) [134,135,136,137]. All of these factors result in the activation of NFκB (nuclear factor kappa B) pathway [138,139,140]; and therefore, both lipotoxicity and glucotoxicity are an important source of free radicals and inflammation. Indeed, it is beyond question that NF-κB signalling plays a key role in regulating the amount of ROS/RNS in the cell [141,142]. Considering that the brain is particularly sensitive to the redox abnormalities, peripheral oxidative stress can affect the induction of neuronal oxidative stress (Figure 2). Especially, it is promoted by the increased BBB permeability under IR/hyperglycaemic conditions. Interestingly, Maciejczyk et al. [93] observed a positive correlation between the brain oxidative damage and the HOMA-IR (homeostasis model assessment of insulin resistance) index, which points at the involvement of peripheral IR in the development of neuronal oxidative stress.

**Figure 3:**
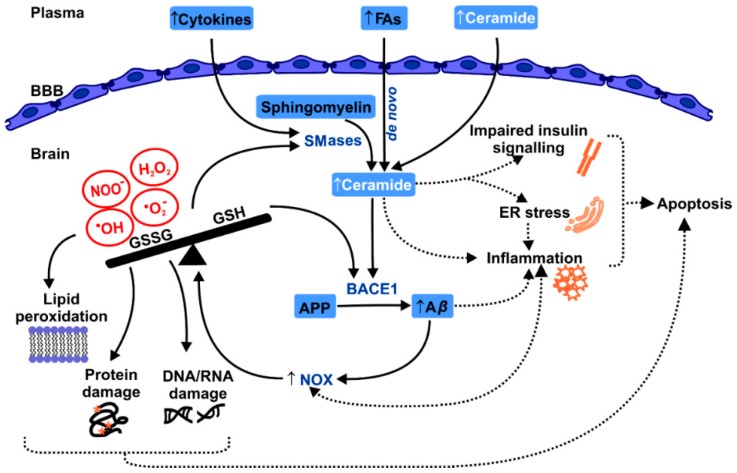
Crosstalk between ceramide, oxidative stress, and the brain insulin resistance. Ceramide plays a key role in the induction of brain IR. Peripheral IR is associated with an elevated ceramide generation. Ceramide like other neurotoxic lipids can pass through the blood-brain-barrier (BBB), contributing to the brain IR via liver-brain axis of neurodegeneration. In IR brain, ceramide can induce neuronal apoptosis and, similarly to Alzheimer’s disease, accumulation of amyloid β-peptides (Aβ). However, a key mediator of Aβ-neurotoxic effects may not be the ceramide but rather oxidative stress. It has been demonstrated that Aβ induces activation of NADPH oxidase (NOX) associated with glutathione (GSH) depletion, lipid and protein oxidation, disturbances in glucose metabolism, as well as mitochondrial abnormalities. Abbreviations: Aβ, amyloid β-peptides; APP, Aβ precursor protein; BBB, blood-brain-barrier; BACE1, β-secretase; ER, endoplasmic reticulum; FAs, fatty acids; GSH, reduced glutathione; GSSG oxidised glutathione; NOX, NADPH oxidase; SMases, sphingomyelinases.

Due to the fact that ceramide mediates IR and can cross the BBB, there is a relationship between peripheral IR and cerebral degeneration [8,45,46]. However, it has been demonstrated that exposure to short-chain ceramide results not only in neuronal IR, but also inflammation, mitochondrial dysfunction, and free radical-mediated damage [8,16,45,70,143]. Indeed, it has been proven that the increase in ceramide level intensifies the activity of caspase-3 and caspase-8 (*via* ↓insulin signalling and ↑inflammation), which leads to the increased production of ROS and induction of oxidative stress [65,144,145]. This mechanism is also postulated in the IR brain (Figure 3).

However, ceramide can also enhance formation of amyloid β-peptides through posttranslational stabilization of β-secretase (BACE1), which stimulates proteolytic modifications of Aβ precursor protein (APP) [60,61,146,147]. It is well known that deposition of Aβ protein plays a key role in the neurodegeneration in AD. Nevertheless, increased Aβ level was also reported in the IR patients [67,148]. Interestingly, a key mediator of Aβ-neurotoxic effects may not be directly related to ceramide but rather to oxidative stress (Figure 3). The resulted Aβ peptide highly up-regulates NADPH oxidase (NOX) generating a large amount of superoxide anions (O_2_^−•^) associated with GSH depletion, mitochondrial abnormalities, and oxidation of lipids, proteins, and nucleic acids [46,60,61,146]. Indeed, a high activity of pro-oxidant enzymes: NOX and xanthine oxidase (XO) was recorded in IR brain, which dramatically enhances consumption of oxygen (during respiratory burst in mitochondria), and therefore, may further induce cytokine activation in the brain inflammatory cells [93,149,150,151]. It has also been demonstrated that in the IR brain expression of cytokines (e.g., IL-1β, IL-6, TNFα), chemokines, and pro-inflammatory enzymes (e.g., COX-2 and iNOS) is significantly increased, not only in the hypothalamus and cerebral cortex, but also in other brain structures such as cerebellum, amygdala and hippocampus [28,102,152,153,154]. It has also been evidenced that IL-1β reduces glucose transport into the cells by inhibiting the expression of IRS-1, whereas TNFα, IL-6 and IL-1β lead to the activation of JNK kinase and κB kinase inhibitor (IKKβ), which decreases brain insulin sensitivity [55,102,152,155]. Interestingly, also AGEs have been shown to stimulate pro-oxidant and pro-inflammatory signalling (e.g., NFκB, JNK, and MAPK), as well as directly enhance NOX activity, which corresponds to ROS overproduction as well as neuronal inflammation [102,116,119]. Additionally, higher formation of ROS can also activate cerebral sphingomyelinases (SMases), which hydrolyse membrane sphingomyelin and further increase the production of ceramide in the brain [46,60,61]. Consequently, oxidative stress may be a critical mediator of ceramide’s neurotoxic effects, which partially explains the relationship between the brain IR and neurodegeneration in Alzheimer’s disease (Figure 3).

As shown in recent studies, also excessive levels of circulating FAs may increase the formation of intracellular ROS in the brain [156,157,158,159]. Interestingly, in these conditions, ROS overproduction results in neuronal toxicity by inducing neuroinflammation and endoplasmic reticulum (ER) stress [16,54,157]. It is suggested that FAs can pass through the BBB and enhance *de novo* synthesis of ceramide, which can explain the induction of brain IR as well as neuronal redox imbalance (Figure 3).

## 7. Mitochondrial Abnormalities in the Brain Insulin Resistance

**Figure 4:**
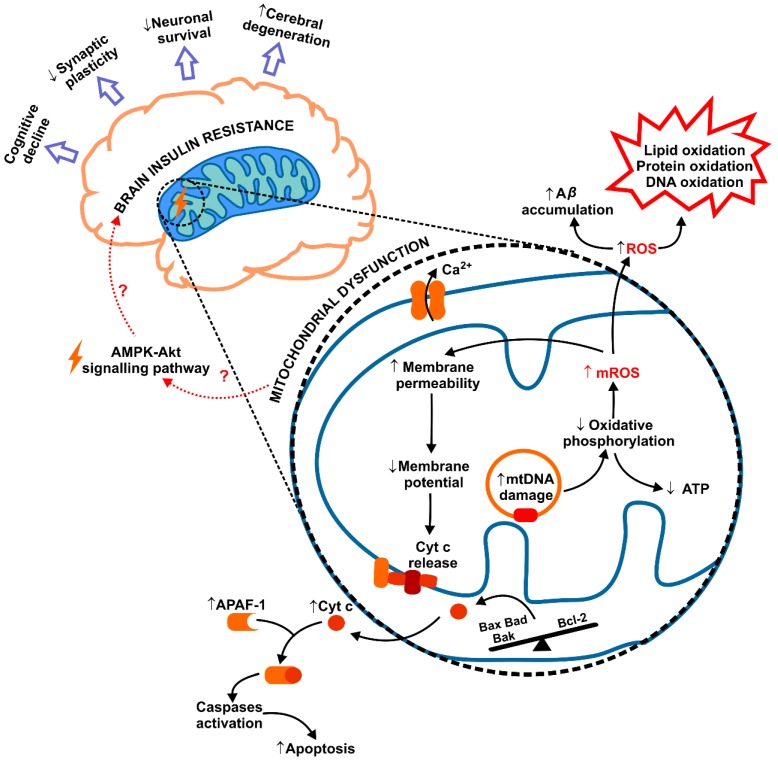
Mitochondrial dysfunction in brain insulin resistance. The results of recent studies indicate the role of mitochondrial abnormalities in the brain IR. Increased production of mitochondrial ROS (mROS), cytochrome c release, as well as functional and ultrastructural changes of mitochondria have been confirmed in IR brain. Interestingly, brain mitochondrial dysfunction and Aβ accumulation may be responsible for disturbances in apoptosis, synaptic plasticity, cognitive decline, and cerebral degeneration in IR patients. Abbreviations: Aβ, amyloid β-peptides; APAF1, apoptotic protease activating factor 1; BAD, Bcl-2-associated death promoter; BAK, Bcl-2 homologous antagonist killer; BAX, Bcl-2-associated X protein; cyt c, cytochrome c; mROS, mitochondrial ROS; mtDNA, mitochondrial DNA; ROS, reactive oxygen species.

Mitochondria play a vital role in energy homeostasis of the cell. In addition to the bioenergetic processes, they also participate in insulin signalling, cell death/survival control, as well as are the main site of ROS generation in the cell during respiratory reactions in mETC. Therefore, it is not surprising that mitochondrial abnormalities are highly related to the development of peripheral IR [5,134,160,161]. It is well known that in IR/obese patients, excessive supplies of glucose and FAs contribute to the higher formation of mitochondrial ROS (mROS) produced as by-products of mETC [136,137,160]. Under these conditions, particularly large amounts of superoxide anion (O_2_^−•^) are formed, which not only oxidizes cell components but also inhibits the activity of glycolytic enzymes [160]. Additionally, the results of recent studies indicate a pivotal role of mitochondrial dysfunction also in the brain IR (Figure 4) [9,28,103,135]. In fact, decreased activity of brain mETC, reduced mitochondrial respiration, mutations in mitochondrial DNA (mtDNA), as well as disturbances in mitochondrial fusion and fission were observed in vitro and in animal models of IR [9,28,103,135,162]. This results not only in reduced ATP synthesis, decreased O_2_ consumption and CO_2_ generation, but also in mROS overproduction and associated redox imbalance [162,163,164]. Furthermore, it was shown that in IR brain, enhanced mROS levels lead to neuronal apoptosis observed as higher expression of pro-apoptotic proteins (Bax, Bad, Bak), lower levels of anti-apoptotic Bcl-2, as well as caspases signalling activation [9,28,103,135]. Indeed, it has been demonstrated that cytochrome c (cyt c) is released following mitochondrial swelling and forms the complex with APAF1 (apoptotic protease activating factor 1), which stimulates the caspase cascades in the brain [7,9,28] (Figure 4).

Several studies reported that mROS overproduction enhances the accumulation of amyloid β-peptides and induces oxidative damage to proteins, lipids and nucleic acids in the IR brain [16,153,165,166]. Interestingly, also the decrease in dendritic spine density and long-term potentiation (LTP) processes were linked to the mitochondrial abnormalities in the IR brain [41,98,167,168,169]. Thereby, the imbalance in mitochondrial dynamics and malfunction of brain mitochondria can be responsible for both disturbances in neuronal apoptosis, as well as synaptic plasticity, cognitive decline, and cerebral degeneration in the IR brain [153,165,166] (Figure 4). Interestingly, in the animal model of Alzheimer disease, mitochondrial abnormalities and the deposition of Aβ peptides are dependent on a degree of cognitive impairment in AD transgenic mice [165]. A similar relationship is also postulated in the IR brain.

It has been also demonstrated that the pharmacological treatment such as anti-diabetic drugs (e.g., biguanide, peroxisome proliferator-activated receptor gamma (PPARγ) agonist, sodium-glucose co-transporter 2 (SGLT-2) inhibitors), hormone therapy (e.g., glucagon-like peptide-1 (GLP-1) and glucose-dependent insulinotropic polypeptide (GIP)), and antioxidant supplementation (e.g., N-acetyl-L-cysteine (NAC), α-lipoic acid (ALA)) could improve not only the whole body metabolic parameters but also the brain’s insulin sensitivity and its mitochondrial function [9,98,163,170,171]. Interestingly, similar benefits were also observed in patients with AD [172,173,174]. However, only few reports indicate that neuronal mitochondrial damage is associated with impaired insulin signalling pathways (e.g., AMPK-Akt) [42], and thus, with induction of the brain IR. Indeed, there is no convincing evidence that the mitochondrial dysfunction predisposes to the induction of the brain IR, and therefore, further investigation is needed to achieve a better understanding of IR pathogenesis in the brain.

## 8. Conclusions

Several studies demonstrated that brain IR is inextricably linked to oxidative stress associated with accumulation of ceramide and protein aggregates, activation of pro-inflammatory cytokines, mitochondrial dysfunction, as well as neuronal apoptosis. It is believed that ROS overproduction occurs due to metabolic abnormalities accompanying peripheral IR and with an impaired mitochondrial activity in the IR brain. Recent studies have also shown that oxidative stress enhances the accumulation of amyloid β-peptides as well as decreases in dendritic spine density and long-term potentiation (LTP) in the IR brain. Therefore, redox imbalance may play a key role in cerebral degeneration, cognitive impairment, and increased incidence of Alzheimer’s disease in IR patients. Although several studies found the relationship between neuronal oxidative stress and the brain IR, it remains unclear whether redox imbalance is a primary cause of brain IR. We must not ignore the fact that the brain oxidative stress may also be the consequence of peripheral/brain IR. Hence, further studies are needed to better understand the role of oxidative stress in the IR brain as well as indicate the benefits of the use of antioxidant supplements.
